# The Homeodomain Protein Ladybird Late Regulates Synthesis of Milk Proteins during Pregnancy in the Tsetse Fly (*Glossina morsitans*)

**DOI:** 10.1371/journal.pntd.0002645

**Published:** 2014-04-24

**Authors:** Geoffrey M. Attardo, Joshua B. Benoit, Veronika Michalkova, Kevin R. Patrick, Tyler B. Krause, Serap Aksoy

**Affiliations:** 1 Yale School of Public Health, Department of Epidemiology of Microbial Diseases, New Haven, Connecticut, United States of America; 2 Institute of Zoology, Slovak Academy of Sciences, Bratislava, Slovakia; National Institute of Allergy and Infectious Diseases, United States of America

## Abstract

Regulation of tissue and development specific gene expression patterns underlies the functional specialization of organs in multi-cellular organisms. In the viviparous tsetse fly (*Glossina*), the female accessory gland is specialized to generate nutrients in the form of a milk-like secretion to support growth of intrauterine larva. Multiple milk protein genes are expressed specifically in the female accessory gland and are tightly linked with larval development. Disruption of milk protein synthesis deprives developing larvae of nutrients and results in extended larval development and/or in abortion. The ability to cause such a disruption could be utilized as a tsetse control strategy. Here we identify and delineate the regulatory sequence of a major milk protein gene (*milk gland protein* 1:*mgp1*) by utilizing a combination of molecular techniques in tsetse, *Drosophila* transgenics, transcriptomics and *in silico* sequence analyses. The function of this promoter is conserved between tsetse and *Drosophila*. In transgenic *Drosophila* the *mgp1* promoter directs reporter gene expression in a tissue and stage specific manner orthologous to that of *Glossina*. Analysis of the minimal required regulatory region of *mgp1*, and the regulatory regions of other *Glossina* milk proteins identified putative homeodomain protein binding sites as the sole common feature. Annotation and expression analysis of *Glossina* homeodomain proteins identified *ladybird late* (*lbl*) as being accessory gland/fat body specific and differentially expressed between lactating/non-lactating flies. Knockdown of *lbl* in tsetse resulted in a significant reduction in transcript abundance of multiple milk protein genes and in a significant loss of fecundity. The role of Lbl in adult reproductive physiology is previously unknown. These results suggest that Lbl is part of a conserved reproductive regulatory system that could have implications beyond tsetse to other vector insects such as mosquitoes. This system is critical for tsetse fecundity and provides a potential target for development of a reproductive inhibitor.

## Author Summary

Female tsetse flies (Diptera: *Glossina*) harbor and give birth to live young. To do this, they nourish their intrauterine larvae with milk secretions. This work focuses upon understanding the regulation of tsetse milk proteins, which are essential for fecundity and are expressed in a temporally and spatially specific manner by pregnant females. We identified the minimal upstream regulatory DNA sequence of the major milk protein gene *mgp1*, which confers tissue specific expression in the female accessory glands of reproductively active flies. This regulatory sequence functions similarly in transgenic fruit flies (*Drosophila melanogaster*) and drives expression of reporter gene products in the adult female accessory gland. Comparison of this regulatory sequence with sequences from other characterized milk proteins indicates that conserved homeodomain transcription factors may be responsible for regulating these genes. Analysis of *Glossina* homeodomain proteins identified an accessory gland/fat body specific factor, *Ladybird late* (*lbl*), which appears to regulate the expression of multiple milk proteins. Reduction of *lbl* levels interferes with milk protein gene expression, which in turn reduces *Glossina* fecundity. These results suggest that milk proteins in *Glossina* are regulated by a conserved regulatory system mediated in part by the homeodomain transcription factor *lbl*. Components of this system could provide a target for development of a tsetse reproductive inhibitor.

## Introduction

Tsetse flies (*Glossina sp.*) are the exclusive vectors of Human African Trypanosomiasis (HAT) and nagana in Sub-Saharan Africa. HAT transmission can be prevented through tsetse population control by baited targets and trapping [1]. These control methods are effective due to the unusual nature of tsetse's reproductive physiology. Tsetse females reproduce via adenotrophic viviparity (the intrauterine retention and nourishment of larval offspring). Each female is capable of producing only 8–10 progeny in their 2–3 month life span [2]. As a result, vector control strategies targeting population reduction can have a significant and quick impact upon population density and disease transmission dynamics.

Viviparity in tsetse entails the protection and nourishment of offspring for the duration of immature (larval) development by the mother [3]. Larval nourishment is supplied as a “milk” secretion that is generated by the accessory gland (milk gland) that empties into the uterus. The tsetse milk gland is orthologous to accessory glands found in association with the female reproductive tract of other insects. Female accessory glands (also referred to as paraovaria or colleterial glands) in insects are adapted in a variety of novel ways to accommodate different reproductive strategies and life histories. In tsetse, milk gland morphology differs from the accessory glands of other insects. The milk gland expands from its connection to the uterus throughout the abdomen as a series of bifurcating tubules that intertwine with the abdominal fat body tissue [4]. The secretory cells of the tubules synthesize and secrete large volumes of milk [5]. At the time of parturition, larvae are almost equivalent in weight to the mother, illustrating the tremendous maternal nutritional investment in each gonotrophic cycle. Development of a control strategy to disrupt lactation in tsetse would be an effective way to reduce the vector population.

Molecular analysis of milk gland secretions has identified Milk Gland Protein 1 (MGP1 - VectorBase accession:*GMOY009745*) as one of the primary protein components of tsetse milk [6]. MGP1 is a member of the lipocalin protein family, which are known for their ability to bind small hydrophobic molecules [7]. Lipocalins are common constituents of lactation secretions in marsupials [8], mammals [9] and are a primary component of the secretion generated by the viviparous cockroach species *Diplotera punctata* [10]. Synthesis of MGP1 in tsetse is exclusive to the secretory cells of the milk gland. Transcript/protein levels correlate with the demand for nutrients by the intrauterine larvae. Prior to larval development there is little to no expression of this gene. Beginning at late embryogenesis/early larvigenesis, *mgp1* transcript and protein levels begin to increase and reach their maximum when the larva reaches its 3^rd^ instar (the time of highest nutritional demand) [6]. Knockdown of *mgp1* by dsRNA treatment reduces female fecundity suggesting that this protein is required for larval development [11].

Transcriptomic analysis of pregnant females and proteomic analysis of milk gland secretions have provided knowledge on the protein constituents of tsetse milk [12]. These proteins include Transferrin (*trf*) [13], Acid Sphingomyelinase (*asmase1*) [14] and nine unique proteins (*mgp2*-10) the function of which is to be determined [12], [15] (see Table S11 in the *Glossina* genome paper) [16]. Milk associated proteins are conserved in tissue and stage specific expression in a manner similar to MGP1 [12]. Identification of the signals and mechanisms coordinately regulating milk protein expression is an important goal in the search for novel vector control targets.

The recent sequencing of the tsetse genome [16] and the advent of next generation high throughput technologies have allowed us to study the pregnancy and tissue specific nature of tsetse's most abundant milk protein gene (*mgp*1), focusing on the role of specific transcription factors that may be associated with the upstream promoter region. We performed our analysis of milk protein gene regulation in tsetse using a combination of classic molecular techniques and genetic analysis in transgenic *Drosophila*. We used *in silico* analysis of genomic data and multiple high throughput datasets coupled with functional studies in *Glossina* and in *Drosophila* to identify candidate homeodomain factors that are apparently involved in the regulation of multiple tsetse milk protein genes. Our results suggest conservation of a reproduction associated gene regulatory mechanism across different taxa (*Glossina* and *Drosophila*) in the context of differing reproductive physiologies. We discuss our findings in light of potential methods to disrupt the reproductive capacity of tsetse as a vector control tool.

## Materials and Methods

### Primers

qPCR primer sequences are found in Table S1. All cloning primer sequences are presented in Table S2 and siRNA sequences in Table S3.

### *Glossina* rearing

The *Glossina morsitans morsitans* colony maintained in the insectary at Yale University was originally established with puparia from fly populations in Zimbabwe. Newly emerged flies are separated by sex and mated at three to four days post-eclosion. Flies are maintained at 24±1°C with 50–55% relative humidity, and receive defibrinated bovine blood every 48 h using an artificial membrane system [17].

### Quantitative expression analysis of *mgp* during parturition

Samples for gene expression analysis during parturition were collected as follows. Female flies pregnant with 3^rd^ instar larvae were collected. Flies were checked every 24 hours and individuals that had undergone parturition during the 24 hour period were collected to stage them in sample groups. Three flies were collected at the following time points, pregnant (3^rd^ instar larva), 0–24, 24–48, 48–72, 72–96, 96–120, 120–144, 144–168, and 168–192 hrs post parturition (pp). RNA and protein were isolated from flash frozen female flies and tissue samples utilizing the standard Trizol (Invitrogen, Carlsbad CA) protocol with a modification to the final step in which the isolated protein pellets are dissolved in cracking buffer (8M urea, 3M thiourea, 1% dithiothreitol (DTT) and 4% CHAPS) [18]. cDNA was prepared from total RNA using the Invitrogen Superscript III kit (Invitrogen). Levels of *mgp1* were determined by CFX Connect Real Time PCR Detection System with SYBR Green Supermix (Bio-Rad, Hercules, CA). The data were analyzed with software version 3.1 (Bio-Rad). All samples were normalized according to *Glossina* tubulin (*tub*) expression levels.

### Western blotting

Western blotting was performed utilizing the protein from the samples described above using previously described antisera and protocols [6], [19].

### *Glossina* genomic data

Genomic sequences and automated annotations were derived from the recently completed *G. m. morsitans* genome [16] and are available at Vectorbase (http://gmorsitans.vectorbase.org/Glossina_morsitans/Info/Index).

### Creation of β-gal enhancer/reporter constructs and *Drosophila* transformation

Two constructs were created using 2 kB and 0.5 kB of the 5′ upstream from the *mgp1* predicted transcription start site. These fragments were PCR amplified from *G. m. morsitans* genomic DNA using Platinum Taq DNA Polymerase High Fidelity (Invitrogen) and sub-cloned into the T-vector cloning vector (Promega, Madison WI). Inserts were excised from the T-vector using SphI and NotI and ligated into the *p-element* vector pPelican [20] between the SphI and NotI sites. This vector includes a *lacZ* reporter gene downstream of the regulatory sequence. The enhancer/reporter constructs were used to transform *w^1118^* flies by p-element mediated transformation via a commercial transformation service (Best Gene: *Drosophila* Injection Services (http://www.thebestgene.com). The transformations generated 4 lines carrying the 2.0 kB construct *mgp1-Bgal-2.0-1-4* and 3 lines carrying the 0.5 kB construct *mgp1-Bgal-0.5-1-3*. *Drosophila* lines were maintained according to standard protocols [21].

### *Drosophila* dissection, ß-galactosidase staining, imaging and activity

Tissues from male and female *Drosophila* were dissected in 1× PBS. Dissected tissues were fixed and stained for β-galactosidase activity with the ß-galactosidase Staining Kit (Mirus, Madison WI) following the manufacturer's protocols.

### Creation of eGFP enhancer/reporter constructs and PhiC homologous recombination mediated *Drosophila* line creation

Flies expressing an eGFP reporter with 509 (*mgp1-egfp-509*), 236(*mgp1-egfp-236*), 112 (*mgp1-egfp-112*) or 13 bp (*mgp1-egfp-13*) nucleotide versions of the *mgp1* upstream sequence were generated utilizing the PhiC homologous recombination transformation system [22], [23]. The 509 bp *mgp1* upstream fragment was PCR amplified from *Glossina morsitans* genomic DNA using Platinum Taq DNA Polymerase High Fidelity (Invitrogen) and sub-cloned into the T-vector cloning vector (Promega). The fragment was excised with SphI and SpeI then ligated into the nuclear EGFP enhancer analysis vector pStinger [20] between the SphI and NheI sites by standard cloning methods. The *mgp1*-pStinger construct was used as a template to amplify the 509, 236, 112 and 13 base pair variants of the *mgp1* promoter-*egfp* fusion constructs. The forward and reverse primers for the constructs were tailed with the AttB40 PhiC recombination site sequence. Amplified constructs were cloned into T-vector and sent for transformation via a commercial transformation service (Best Gene: *Drosophila* Injection Services (http://www.thebestgene.com). Constructs were injected into the *Drosophila* line genotype y^1^ w^*^; P[attP.w^+^.attP]JB53F (Bloomington Stock Center #27386). The AttB integration site is 53F8, 2R:12985015 [22]. Surviving injected flies were crossed with CyO/Sco 2^nd^ chromosome balancer flies and screened for loss of eye color as a negative marker of transgene insertion. *Drosophila* lines were maintained according to standard protocols [21].

### Visual and quantitative analysis of EGFP transgene expression in transgenic *Drosophila*

RNA was isolated from flies using Trizol (Invitrogen). cDNA was prepared from total RNA using the Invitrogen Superscript III kit (Invitrogen). Transgene transcript levels were quantified by qPCR with iQ SYBR Green Supermix using *egfp* specific primers and the CFX Connect Real Time PCR Detection System (Bio-Rad). Data was analyzed using software version 3.1 (Bio-Rad). All treatments were normalized according to *Drosophila beta-tubulin* expression levels using gene specific primers and carried out in triplicate. Visual inspection of transgene expression was performed by tissue dissection in 1× PBS followed by fluorescent microscopy using a Axio Cam dissecting scope equipped with a X-cite series Q fluorescence system (Zeiss Microscopy & Image Analysis, Thornwood, New York).

### Nutritional deprivation and stage specificity experiments on transgenic *Drosophila*

Stage specific quantification of *egfp* transgene expression was performed as described above with total RNA from 3 independent groups of larval, pupal, and 3–5 day old male and female flies from the *mgp1-egfp-509* line. 3–5 day old adult females from the *mgp1-egfp-236*, *mgp1-egfp-112* and *mgp1-egfp-13* lines were also collected and analyzed in the same manner to compare transgene expression between the lines.

Nutritional deprivation experiments were performed by maintaining the *mgp1-egfp-509* line on either complete (yeast extract 100 g/L, sucrose 100 g/L, Agar 27 g/L, Methlyparaben: 30 ml of 100 g/L, Proprionic acid 3 mL/L) or minimal media (yeast extract 12.5 g/L, sucrose 12.5 g/L, Agar 27 g/L, Methlyparaben: 30 ml of 100 g/L, Proprionic acid 3 mL/L) in 25×95 mm polystyrene vials. Egg deposition was monitored daily for each group. Parallel treatments were run to quantify *egfp* levels in stressed flies using the qPCR methods described above.

### *In silico* transcription factor binding site prediction

Putative transcription factor binding sites were predicted within the *mgp* upstream regulatory region using the “MatInspector” program from Genomatix (http://www.genomatix.de/) [24]. Upstream sequences from other characterized and predicted milk proteins were derived from the draft *Glossina* genome (www.vectorbase.org) [16]. The sequences used were from the 124 bp *mgp1* (*GMOY009745*) critical region, *trf* (*GMOY004228*), *asmase1* (*GMOY002246*), *mgp2-10* (*GMOY012368*, *GMOY012125*, *GMOY001342*, *GMOY012016*, *GMOY001343*, *GMOY012016*, *GMOY012369*) genes. 500 bp from the predicted transcription start site of each gene was used in a comparative analysis. This analysis was also performed using MatInspector.

### *Glossina* homeodomain factor identification and annotation

Predicted *Glossina* homeodomain sequences were identified from the *Glossina* genome [16]. Protein sequences from the 104 known *Drosophila* homeodomain factors were used to search the *Glossina* genomic assembly and the *de novo* assembly from [12] by tBLASTn search. All resulting hits with scores lower than 1×10^−10^ were collected and searched against the NCBI database by BLASTx search. Sequences were then annotated with nomenclature from the highest scoring hit result.

### RNA-seq analysis of differential gene expression during pregnancy

We have recently completed a transcriptome study focusing on differences between pregnant/lactating or dry flies (non-lactating) [12]. Raw sequence data are available from the sequence read archive at NCBI. We utilized the RNA-seq reads from that study to compare the transcript abundance of *Glossina* homeodomain proteins in lactating and dry flies. RNA-seq data were mapped directly to the *Glossina* homeodomain genes utilizing CLC Genomics Workbench (CLC bio, Cambridge, Massachusetts). Normalized gene expression was calculated as RPKM [25]. Statistical differences of RPKM values between samples were determined by Kal's test following Bonferroni correction [26]. Statistical significance was determined at P<0.05 and a two-fold higher or lower transcript abundance in lactating flies was utilized to determine genes of interest [27].

### Homeodomain expression analysis

Five candidate *Glossina* homeodomain genes were analyzed by qPCR based tissue and stage specific expression analysis. Tissue-specific samples were acquired from females harboring 2nd instar larva. qPCR was conducted as before and transcript levels were normalized to *tubulin*.

### *lbl* siRNA knockdown and phenotype analysis

Functional analysis of the ladybird late homeodomain factor was performed using synthesized siRNAs homologous to the *Glossina ladybird late* ortholog. siRNAs were designed by web-based tools (IDT, Coralville, IA) and ordered commercially (IDT). The siRNA consists of two Duplex sequences for *lbl*. Control siRNA molecules were designed as complementary to green fluorescent protein (GFP) mRNA (IDT, Cordville, IA). Concentration of each siRNA was adjusted to 700–750 ng/µl in PBS with a Nanodrop spectrophotometer. Mated female flies were injected with 1.5 µl siRNA 6–8 d after adult emergence. siRNA was shown to have no discernible effect on larval transcripts [28]. Expression levels were determined by qPCR (as described; normalized to *tubulin*) 5 d after injection for *lbl* and 8 d after injection for *mgp1*.

## Results

### Transcriptional regulation of *mgp1* during pregnancy

**Figure 1:**
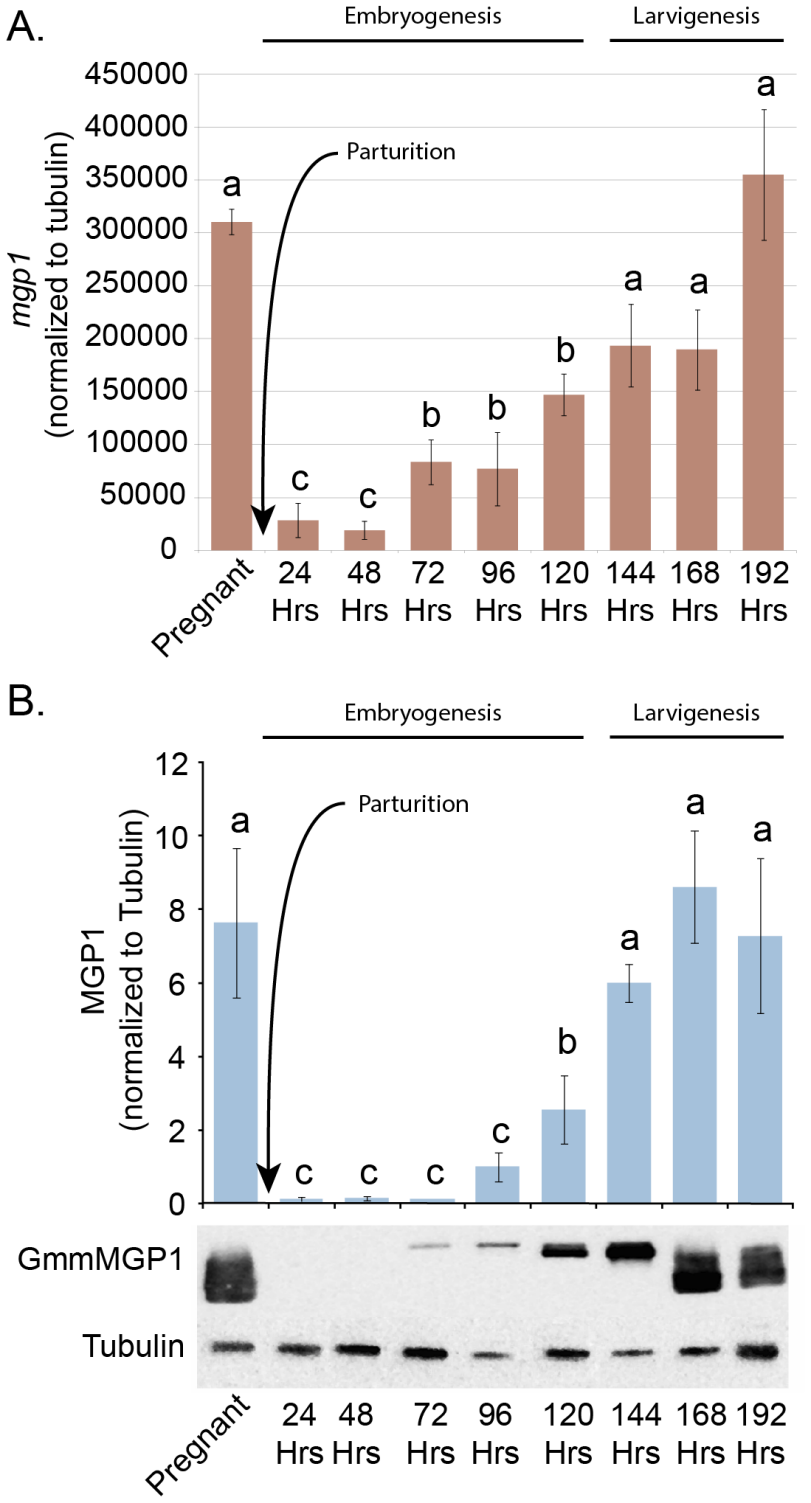
Pre and post parturition timecourse of *mgp1* transcript and protein levels in *Glossina*. **A.** Quantitative PCR analysis of *mgp1* transcript levels just prior to parturition and in 24 hour periods after parturition. Pregnant *Glossina* flies carrying 3^rd^ instar larvae were collected and staged by time of parturition within 24 hour windows. The values represent the mean *mgp1* transcript level from 3 individual flies at each point. Error bars represent standard error. All qPCR data was normalized to tubulin. Statistical notation: a = not significantly different than pregnant; b = significantly different from pregnant (P-value <0.05 by students *t*-test); c = significantly different from pregnant (P-value <0.01 by students *t*-test). **B.** Western blot analysis of MGP1 levels in flies from the same samples as described in A. Values represent quantification of the mean signal intensity from 3 individuals for each point and are normalized to tubulin levels. Statistical notation is as described above.

Transcript abundance of the *mgp1* gene is related to intrauterine larval development. Previous expression data for *mgp1* was based upon samples staged by female reproductive physiology and larval development [15]. Based upon these data, *mgp1* transcripts appear to decrease after larval deposition (parturition) and increase again at the onset of intrauterine larval development in the next pregnancy cycle. To obtain a high resolution temporal analysis of milk protein gene expression during this period, pregnant flies were collected and synchronized by time of parturition in 24 hour intervals rather than by pregnancy status. Transcript levels from these samples were quantified by qPCR analysis, and protein expression was determined by Western analysis. The results show that *mgp1* transcript abundance and protein levels undergo a precipitous drop after parturition and reach their lowest levels at 24–48 hours post-parturition (Figure 1A and 1B). Transcript and protein levels begin to increase again between 48–72 hours post parturition and continue to increase to the last time point in the series at 168–192 hours post parturition when there is a 2^nd^ instar intrauterine larva in the uterus.

### Analysis of *mgp1* promoter functions in transgenic *Drosophila*

Analysis of the regulation of *mgp1* expression required the development of genetic tools that facilitate the observation of *Glossina* promoter function *in vivo*. To accomplish this, we leveraged the genetic tools available in *D. melanogaster* to perform an *in vivo* analysis of the *mgp1* promoter. *Drosophila* is related to *Glossina* as both are members of the dipteran “Higher Flies” (Brachycera) suborder.

The transformation constructs were developed using the pPelican enhancer/reporter plasmid [20]. The plasmid contains a *β-galactosidase* (*β-gal*) reporter gene and the promoter/reporter fusion is flanked by gypsy insulator sequences to prevent the influence of local regulatory elements. Transformation was accomplished by p-element transposition. Reporter expression was visualized by staining for β-gal activity in dissected transgenic *Drosophila* tissues. Two constructs were created including either 2 kB or 0.5 kB sequence of the 5′ upstream from the predicted *mgp1* transcription start site (Figure S1A). Transformation of these constructs resulted in the production of four transgenic lines for the 2.0 kB construct (*mgp1*-β-*gal-2.0-1 to -4*) and three transgenic lines for the 0.5 kB construct (*mgp1*-β-*gal-0.5-1 to -3*).

**Figure 2:**
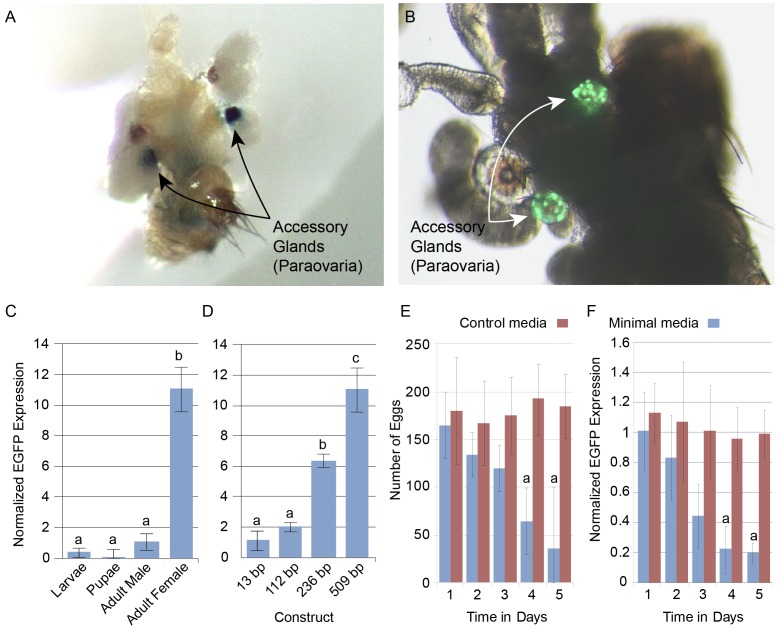
*mgp1* driven reporter expression in transgenic *Drosophila*. Tissue and stage specific expression of *mgp1* driven *ß-gal* and *egfp* reporter genes. All qPCR analyses normalized to Drosophila *tub*. Error bars represent standard error. Letter groups (a,b,c) represent significant statistical differences of P-value <0.05 by students t-test. **A.** Accessory gland specific staining of transgenic Drosophila reproductive tissue (*mgp1- β-gal-2.0*). **B.** Florescent microscopy of accessory gland specific egfp expression in transgenic Drosophila (*mgp1-egfp-509*). **C.** qPCR analysis of sex/stage specific *egfp* levels in transgenic line *mgp1-egfp-509*. Samples represent the average of three groups of 20 flies. **D.** qPCR analysis of *egfp* levels in 3–5 day old mated adult females from the *mgp1-egfp-509*, *mgp1-egfp-236*, *mgp1-egfp-112* or *mgp1-egfp-13* lines. Samples represent three groups of 20 flies. **E.** 3–5 day old transgenic Drosophila (509 bp-*mgp1/egfp*) were reared on control or nutrient deficient media. Data represent the mean eggs/day from five groups of 60 flies. Statistical notation: a = statistical difference between minimal and control media (P-value <0.05 by students t-test). **F.** qPCR analysis of *egfp* levels in *mgp1-egfp-509* flies reared on control or minimal media. Data represents the mean *egfp* transcript level from three groups of 20 flies. Statistical notation: as in E.

In tsetse, *mgp1* expression is specific to the milk gland of adult female flies [11]. We analyzed different tissues from both *mgp1*-β-*gal-2.0* and *mgp1-* β-*gal-0.5* lines with *β-gal* staining to understand the sex and tissue specific nature of the reporter gene expression. Transgene expression was exclusive to the accessory glands (paraovaria) of the female reproductive tract in both transgenic lines (Figure 2A) while this staining pattern was not observed in control *Drosophila*. Background staining was observed in the uterus, midgut and in specific cells along the midline of the dorsal abdominal surface in both sexes. This background staining pattern was also observed in untransformed control flies suggesting that this endogenous *β-gal* activity may result from populations of bacteria resident within the fly. The specific staining observed in the accessory glands of females suggests that regulatory mechanisms and factors driving sex and tissue specific expression of *mgp1* in tsetse are conserved in *Drosophila*. It also demonstrates that the transcriptional regulatory elements required for sex and tissue specific expression are contained within the 0.5 kB upstream region of the *mgp1* gene.

### Transgenic analysis of the 0.5 kB *mgp1* regulatory region

To identify the minimal region required for tissue and stage specific gene expression by the *mgp1* promoter, additional transgenic *Drosophila* lines were generated. To eliminate the confounding issues of background staining with endogenous β-gal activity and variation of transgene expression due to position effect, an alternative transformation strategy was utilized. The new transgenic lines were generated using the PhiC recombination system [22], [23]. This system allows for the stable incorporation of transgenes into a specific genomic locus via homologous recombination, thereby eliminating position effect derived variation in expression between lines. To enhance transgene detection and reduce background, the *β-gal* reporter gene was replaced with the nuclear specific enhanced green fluorescent protein (*EGFP*) reporter gene from the pStinger p-element transformation vector. We generated four transgenic lines, which include either 509 (*mgp1-egfp-509*), 236 (*mgp1-egfp-236*), 112 (*mgp1-egfp-112*) or 13 bp (*mgp1-egfp-13*) segments corresponding to the *mgp1* regulatory region upstream of the start site (Figure S1B).

All life stages and tissues from these lines were examined by florescent microscopy for nuclear specific EGFP expression. Microscopic analysis of EGFP florescence revealed reporter gene expression is only detectable within the accessory glands of reproductively active (undergoing oogenesis and ovulation) females from the *mgp1-egfp-509* line. This is almost identical to the observations in the β-gal expressing lines (Figure 2B). Of the four lines, the *mgp1-egfp-509* and *mgp1-egfp-236* lines were positive for female accessory specific EGFP expression by visual screening, while EGFP expression in *mgp1-egfp-112* and *mgp1-egfp-13* bp constructs was undetectable by visual observation. These results suggest that the minimal region for tissue specific expression and basic promoter function lies within the 236 bp upstream of the *mgp1* transcription start site. The 124 bp differential region between the *mgp1-egfp-236* and *mgp1-egfp-112* lines appears to carry a transcriptional response element critical to the function of this promoter as a whole.

Quantitative analysis of *egfp* expression during different developmental and sexual stages in *mgp1-egfp-509* reveals that the transgene is only significantly expressed within sexually mature adult females with only background levels present in larvae and males (Figure 2C). Comparison of *egfp* expression between the four lines confirms the results we obtained by microscopy analysis. Expression of the transgene is highest in *mgp1-egfp-509* (Figure 2D). Transgene expression in *mgp1-egfp-236* shows that the overall transcript level is significantly lower than that observed in *mgp1-egfp-509*. However, *mgp1-egfp-236* maintains the sex and tissue specific characteristics of the *mgp1-egfp-509*. Transgene expression in *mgp1-egfp-13* and *mgp1-egfp-112* was equivalent to levels observed in male flies suggesting that regulatory function is compromised in these lines.

### Nutritional and/or reproductive regulation of the *mgp1* regulatory region

The reproductive cycle in *Drosophila* differs from that of tsetse in that oogenesis and embryo deposition occur at a steady rate once the female is sexually mature and mated as opposed to the defined cycles of oogenesis, embryogenesis and larvigenesis observed in tsetse. Nutritional stress in *Drosophila* results in reduction or termination of oogenesis. In the absence of appropriate nutritional stimulation yolk protein gene expression and oocyte development cease. In some cases developing oocytes may undergo apoptosis and reabsorption [29], [30]. To determine if a nutritionally induced reduction in oogenesis and ovulation would influence transgene expression, 3 day old mated female *mgp1-egfp-509* flies were put on a minimal media diet followed by daily observation of egg deposition and *egfp* transcript levels. Maintenance on minimal media relative to complete media resulted in a significant reduction in egg production in the *mgp1-egfp-509* flies within 5 days (Figure 2E). Quantitative analysis of reporter *egfp* expression in flies maintained on minimal media during the 5 day time period shows a direct correlation between transgene expression and egg deposition over time (Figure 2F). These results correlate the down regulation of gene expression activity within the accessory gland with reduced nutritional status/oogenesis. Whether the gland is responding to reduced nutritional stimuli or reduced reproductive stimuli is not yet understood.

### *In silico* response element predictions for the *mgp1* 0.5 kB regulatory region and comparative analysis with other tsetse milk proteins

We next set out to predict transcription factor binding sites that may lie within the 0.5 kb promoter region of *mgp1* responsible for the sex and tissue specific expression profile we observed in both taxa. Analysis of the 0.5 kB regulatory sequence using transcription factor binding site prediction software resulted in a total of 41 predicted binding sites from a variety of factors. The matrices used in the identification included those for known insect transcription factors and eukaryotic basal promoter elements. To reduce the number of candidate factors, we narrowed our analysis to the 124 bp region between lines, *mgp1-egfp-236* and *mgp1-egfp-112*, which is required for sex and tissue specific function. This analysis reduced the number of predictions to 11 candidate binding sites. The sites predicted include generic *Drosophila* homeodomain binding sites, including (DHOM: 2 sites), OVO transcription factor (DOVO: 1 site), Iroquois factor group (IRXF: 1 site), Tailless (DTLL: 1 site), Abdominal B (ABDB: 1 site), Paired homeodomain factors (PRDH: 2 sites), Dead Ringer (DRIF: 1 site), Giant (DGTF: 1 site) and Heat shock factors (DHSF: 1 site).

**Figure 3:**
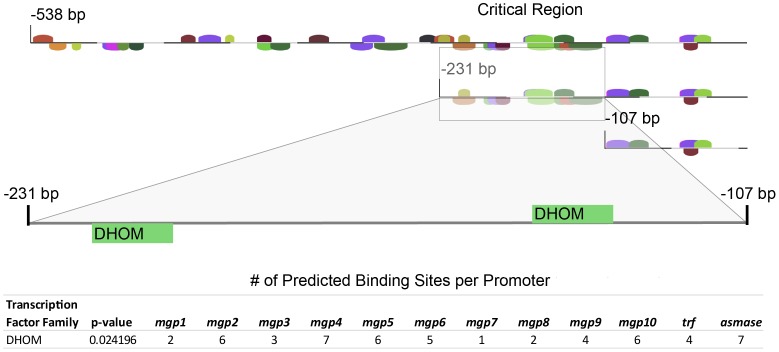
Schematic of the 124*mgp1* regulatory sequence required for tissue and stage specific transgene expression. This schematic represents a scale model of *in silico* predicted homeodomain (DHOM) binding sites. The associated table lists the number of DHOM binding sites predicted within the 500 bp upstream sequence from the 11 other milk protein genes and the associated p-value of these findings.

Previous characterizations, annotation of the *Glossina* genome [16] and RNA-Seq based analysis of lactating and non-lactating flies has identified 12 milk protein genes that function in the lactation process in tsetse, including *mgp1*, *mgp2-10*, *trf* and *asmase1* [12]. Comparative analysis of the 124 bp *mgp1* region critical for transgene expression in *Drosophila* with the 500 bp sequence upstream of the transcription start site of these genes predicts that only homeodomain protein binding sites (DHOM) are common between all 12 sequences (p-value: 0.0241) (Figure 3). The only other site in common between these promoters is that of the TATA binding protein (TATA box).

### Annotation of *Glossina* homeodomain proteins

To identify homeodomain type regulatory factors that may recognize the conserved DHOM sites, we annotated and collated all sequences in *Glossina* bearing homology to the 106 annotated *Drosophila* homeodomain proteins. This was accomplished by tBLASTn search of the *Glossina* genome assembly [16] and a *de novo* assembly of RNA-seq libraries with *Drosophila* homeodomain protein sequences. All significant hits were submitted to a BLASTx comparison against the NCBI protein database. This analysis predicted a total of 96 putative tsetse homeodomain protein orthologs within the current genome assembly (see Table S36 in the *Glossina* genome paper).

Functional analysis of all candidate homeodomain factors was prohibitive. To reduce the number of targets, the expression profile of the 96 homeodomain genes were screened by RNA-Seq analysis using the available Illumina sequencing data from lactating and non-lactating flies [12]. To identify differential transcript abundance between the two physiological states, we selected genes showing a relative transcript difference of greater than 2 fold higher or lower between samples, and sequence representation by at least 500 reads between libraries. Based upon these criteria, our analysis identified 5 homeodomain genes; 3 with high transcript abundance *nubbin* (*nub* – VectorBase accession: *GMOY012175*), *teashirt* (*tsh* – VectorBase accession: *GMOY011052*) and *vismay* (*vis* – VectorBase accession: *GMOY000857*) and 2 with lower transcript abundance *ladybird late* (*lbl* – VectorBase accession: *GMOY004068*), and *pox meso* (*poxm* – VectorBase accession: *GMOY002525*) (Table 1).

**Table 1:** RNA-seq identification of lactation associated homeodomain genes.

Gene Name	Vectorbase Feature ID	Fold Change (normalized values)	Kal's Z-test: 48 vs 192 original values - Test statistic	Kal's Z-test: 48 vs 192 original values - P-value	48 Post-Parturition - # of Normalized reads	192 hrs Post Parturition - # of Normalized reads
nubbin (nub)	GMOY012175	6.62	33.71	0	299	1980.3
teashirt (tsh)	GMOY011052	2.74	32.21	0	1869.4	5127
vismay (vis)	GMOY000857	2.41	41.40	0	4290.4	10347.4
ladybird late (lbl)	GMOY004068	−2.07	−22.89	1.42E-14	2279.6	1099
pox meso (poxm)	GMOY002525	−2.24	−23.40	1.26E-14	2138.2	950.4

RNA–seq statistics comparing the differential expression of putative tsetse homeodomain proteins between transcript datasets from lactating and non-lactating flies. The fold change between samples was tested for significance by Kal's Z-test analysis.

### Tissue specificity of homeodomain factors

**Figure 4:**
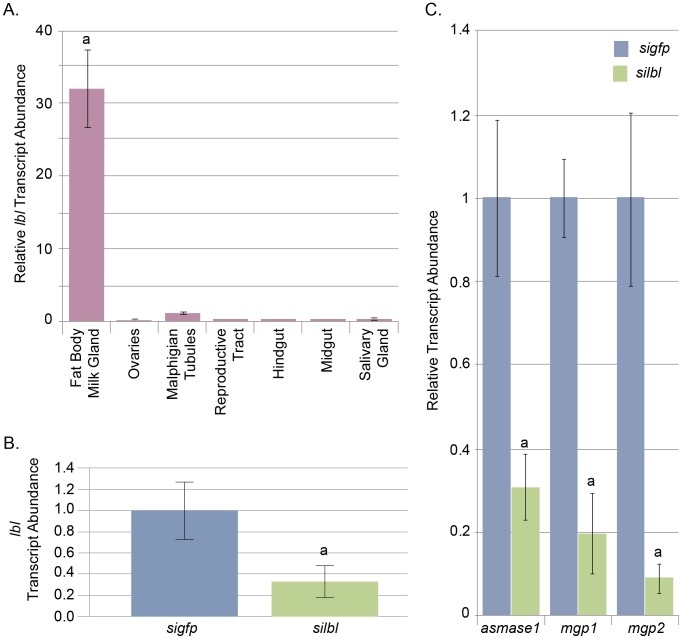
Tissue specificity and siRNA analysis of the *lbl* gene in *Glossina*. All qPCR analyses normalized to Tsetse *tub*. Error bars represent standard error. **A.** qPCR analysis of *lbl* tissue specificity. Samples represent 3 replicates of tissues isolated from 5 individuals. Statistical notation: a = significant difference between groups (P-value <0.05 by students *t*-test). **B.** qPCR analysis of tsetse *lbl* abundance 4 days post *sigfp* (control) or *silbl* injection. Statistical notation: a = significant difference between groups (P-value <0.05 by students *t*-test). **C.** Relative levels of *asmase*, *mgp1* and *mgp2* following injection of *sigfp* (control) or *silbl*. Statistical notation: a = statistical difference from control (P-value <0.01 by students *t*-test). All flies were injected 5–6 days after adult emergence and tested at 11–13 days for *lbl* suppression and 16–17 days for *asmase*, *mgp1* and *mgp2* interference.

Given the tissue specific nature of expression for *mgp1* (and the other milk proteins), homeodomain factors specific to that tissue were of primary interest. We performed a qPCR based expression analysis of the five candidate factors using RNA from different tissue samples obtained from lactating female flies. The expression patterns were unique for each gene. Of the genes analyzed, only the Ladybird Late gene (*lbl*) displayed a milk gland/fat body specific expression pattern. The fat body and milk gland are grouped together due to their interconnected nature which makes separating them by dissection next to impossible. Transcripts for *lbl* were ∼30 fold higher in the milk gland/fat body tissue than in any other tissue (Figure 4A). The other four factors were found in multiple tissues at different levels of transcript abundance (Figure S2).

### Functional analysis of LbL by siRNA knockdown

To determine *lbl* involvement in milk protein gene regulation, siRNA was utilized to perform gene knockdown analyses. Flies were treated with siRNAs against *lbl*, or *gfp* (control). The siRNA injections resulted in significant reductions in target gene transcript abundance. The levels of *lbl* were reduced to ∼30% of controls (Figure 4B). Following siRNA treatment, transcript abundance of the *mgp1*, *mgp2* and *asmase1* genes was measured by qPCR. The knockdown of *lbl*, resulted in a significant decrease of *mgp1*, *mgp2* and *asmase* transcript levels relative to the *GFP* controls (Figure 4C). These data indicate that the *lbl* homeodomain protein functions as a positive regulator of the *mgp1* gene either directly or indirectly.

### Phenotypic effects of *lbl* knockdown on tsetse fecundity

**Figure 5:**
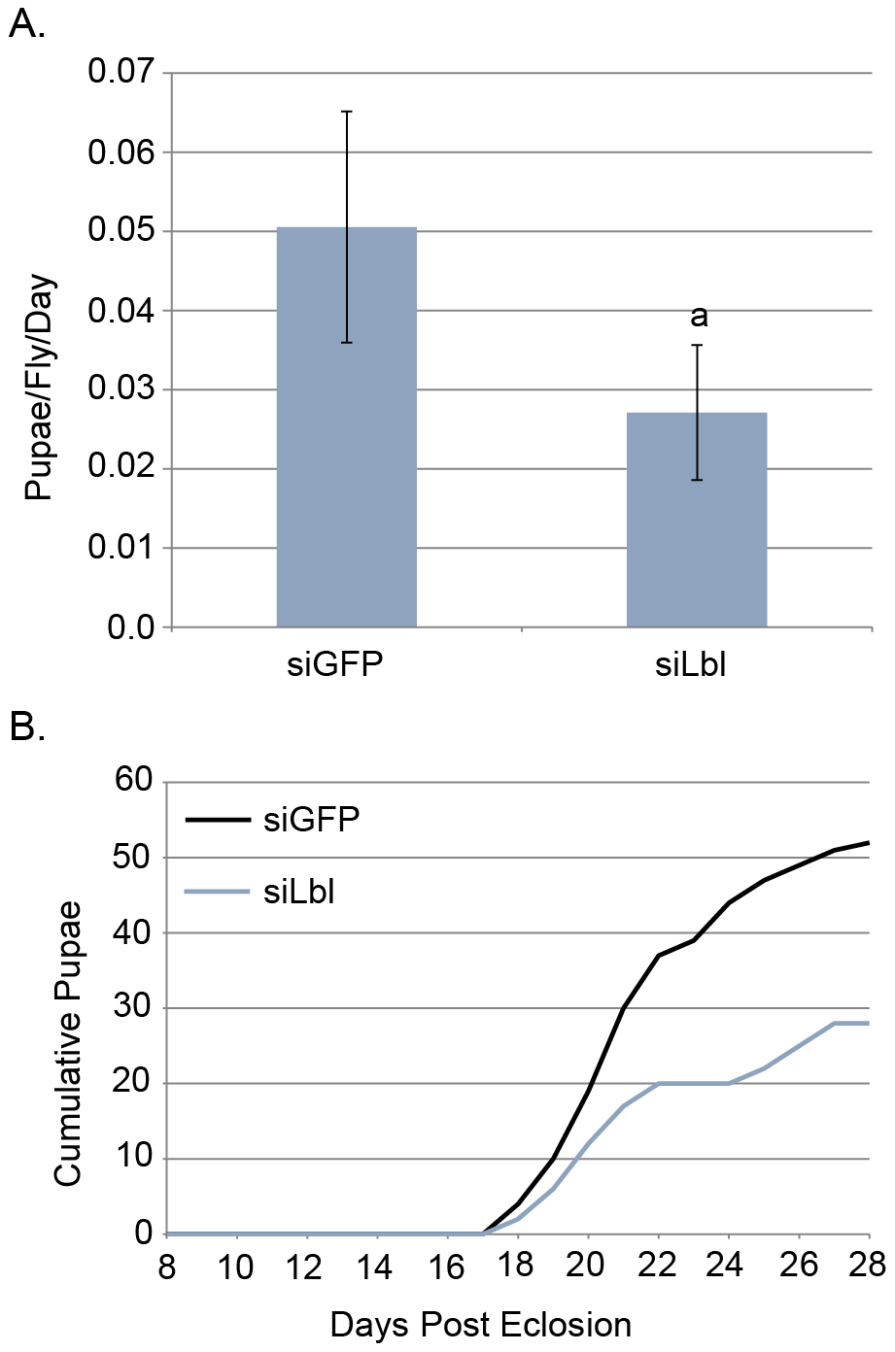
Fecundity effects of *lbl* knockdown. 2 groups of 50 flies were injected with either *sigfp* or *silbl* at 7 days post eclosion and monitored daily for mortality and pupal deposition. **A.** Rate of larval deposition per fly per day after injection of either *sigfp* or *silbl*. Error bars represent standard error. Statistical notation: a = statistical difference between groups (P-value <0.05 by students t-test). **B.** Cumulative count of larval deposition post injection over the course of the first gonotrophic cycle.

We monitored the larviposition of *siLbL* treated mated females over the first gonotrophic cycle. The *lbl* knockdown flies showed a significant reduction in the average rate of larviposition per fly per day relative to controls (Figure 5A). Cumulative larval deposition rates between the control and *lbl* knockdown group revealed that the *lbl* group birthed about half as many larvae as the controls (Figure 5B). The reduction in fecundity in these flies is likely due to the reduced level of milk protein gene expression resulting from the knockdown of the *lbl*. This results in nutritional deprivation of developing larvae, larval death and abortion. This knockdown does not result in a complete disruption of tsetse fecundity in terms of the number of larvae developed per female; however we believe that this is due to incomplete penetrance of our injectable siRNA system in *Glossina*.

## Discussion

The lactation process and the milk proteins are indispensable for intrauterine progeny development in tsetse. Recent transcriptomic/proteomic/genomic analyses in tsetse have provided a comprehensive understanding of the protein constituents of tsetse milk [12], [16]. These proteins act as complete amino acid sources, lipid emulsification and transport agents, providers of enzymatic function aiding larval digestion, transporters of micronutrients/small molecules and regulators of immune function [6], [11]–[15], [31]–[33]. The MGP1 protein is one of the most abundant constituents of tsetse's milk and critical to larval health [6], [11]. Transcript abundance of the *mgp1* gene is tightly associated with the pregnancy status of the female and is restricted to the secretory cells of the milk gland.

This pattern of regulation optimizes the utilization of resources for milk protein production by coordinating gene expression with pregnancy status and nutritional demand by intrauterine offspring. The other milk protein genes display almost identical expression profiles suggesting that these proteins are regulated by the same underlying system that controls *mgp1*. Our studies here indicate that Lbl is responsible for driving the tissue and stage specific expression of the *mgp1* gene and most likely the other milk proteins in tsetse. Furthermore our results show that this tissue specific regulatory mechanism is conserved between *Glossina* and *Drosophila* and that Lbl likely regulates expression of the accessory gland products in the adult female *Drosophila*. The mechanism by which this system monitors pregnancy and larval nutritional demand of tsetse remains unknown and is a target of ongoing study. The powerful genetic capabilities of the *Drosophila* transgenic system will promote these studies and furthermore open up investigations into the little-known biology of insect reproductive physiology.

In tsetse, the milk gland has been a recent focus of study; however less is known regarding the function(s) of female accessory glands in *Drosophila*. Tsetse's physiological adaptations to accommodate viviparous reproduction are unique; but the tissues such as the milk gland and uterus in tsetse are derived from common elements of the female insect reproductive tract. Female accessory gland tissues perform essential functions in insect reproduction. Accessory gland secretions are associated with fertilization, antimicrobial activity, lubrication, embryo adhesion, uterine muscle contraction, defensive secretions and physical protection of embryos. Cockroaches and Mantids (both members of the Dictyoptera clade) have modified glands which produce 2 substances (one from the left and one from the right gland) which combine in the oviduct to generate an ootheca that encases their eggs and hardens into a protective coating [34], [35]. The kissing bug (*Rhodnius prolixus*) uses these glands to produce an adhesive secretion to glue eggs to substrate [36].The accessory glands of many social non-reproductive castes of female hymenopterans have been adapted from reproductive organs to form defensive organs which secrete venom. The conservation of the female accessory regulatory system may extend beyond the higher Diptera, and could be conserved throughout the arthropod lineage as a regulator of glandular gene expression in response to reproductive events. This finding would have implications for other disease vectors such as mosquitoes where little information about accessory gland function is available.

The recent release of the *Glossina* genome sequence [16] and the availability of high throughput sequencing analysis were essential for the analysis of the milk protein promoters, and the identification and screening of the transcription factors we describe here. These *in silico* analyses led us to identify the homeodomain family of transcription factors and allowed us to narrow down our search from a field of 96 potential factors to a handful of 5 factors. Homeodomain proteins are a conserved family of transcription factors characterized by the presence of a helix-turn-helix DNA binding domain called the homeodomain [37], [38]. These factors are associated with coordination of body plan organization during development in metazoan organisms [39]. While the function of some of these proteins is well understood within the context of embryonic development, little is known of their function on transcriptional regulation within mature organisms. In addition, a number of homeobox genes have been identified based upon DNA binding domain conservation, yet little is known as to their functions and if the functions are orthologous between organisms [40].

Here, tissue specific expression profiling and RNAi analysis led us to the Ladybird Late homeodomain factor (Lbl). This factor appears critical to the expression of multiple milk protein genes in tsetse based upon our knockdown experiments. The 236 bp tsetse promoter region was sufficient to drive the synthesis of a reporter gene in an accessory gland specific manner in female *Drosophila*. The conservation of regulatory sequence function in tsetse and *Drosophila* suggests that LbL performs orthologous reproductive functions in both species.

The *lbl* gene was first discovered with its paralog *ladybird early* (*lbe*) during a search for novel homeodomain proteins [41]. This factor and most of the characterized homeodomain proteins are primarily associated with developmental functions in immature organisms. Research on the *ladybird* genes in *Drosophila* have linked these genes with embryonic heart cell precursor diversification [42], embryonic muscle precursor cell diversification (specifically myogenesis in appendages) [43], [44] and embryonic neuroblast diversification [45]. Analysis of *ladybird* gene regulation suggests that the two genes are regulated by the *wingless* (*wg*) signaling pathway and may in turn regulate wingless expression in a feedback loop [46]. This is the first association of Lbl function with adult regulatory processes. The data presented here indicate that Lbl is part of a system that either directly or indirectly regulates gene expression in female accessory tissues. The details of the other components of this system and the reproductive cues it responds to are unknown. The association of *lbl* with *wingless* signaling during development hints at the possibility of the *wingless* pathway as a regulator of reproduction associated gene regulation in adults [46].

The similarity in function of the *mgp1* regulatory sequence between tsetse and *Drosophila* coupled with the ease of transgenesis in the fruit fly system allows us to use *Drosophila* as a surrogate in which to study female accessory gland gene expression. This promoter in combination with GAL4 mediated systems can enable *in vivo* tissue and stage specific knockdowns and/or ectopic expression experiments in *Drosophila* in order to identify components of the signaling system regulating Lbl activity. Flies carrying *mgp1-GAL4*/*mgp1-EGFP* fusions can be crossed with the available *UAS*-RNAi lines [47] of genes of interest (such as components of the *wg* pathway) to perform knockdowns quickly and efficiently. The presence of the *mgp1-EGFP* reporter will allow for rapid visual assessment of knockdown phenotypes. The findings from *Drosophila* can then be translated back into tsetse by RNAi experiments.

These findings are an important step in identifying the pathways, factors and signals regulating milk gland/accessory gland function in insect reproduction. Milk production in *Glossina* is essential for reproductive success and provides an important molecular target for the development of an insect specific reproductive inhibitor. Further analysis of mechanisms governing this system will provide important data towards controlling sleeping sickness as well as broadening the understanding of insect reproductive physiology. The knowledge gained from this work can be put towards the development of an insect specific inhibitor which disrupts milk protein production in tsetse. A substance such as this could be utilized on targets, traps and livestock as a non-toxic alternative to pesticides to reduce tsetse populations and decrease the trypanosome transmission threat.

## Supporting Information

Figure S1 **Schematic of** ***mgp1*** **-reporter fusion constructs for** ***Drosophila*** **transformation.** **A.** 2.0 and 0.5 kB mgp1 pPelican based transformation constructs. Constructs include 2.0 and 0.5 kB promoter fragments driving a β-gal reporter flanked by gypsy insulator sequences. The construct also includes the white transformation marker and is flanked by p-element sequences. **B.** 509,236,112 and 13 bp mgp1 pStinger based recombination constructs. The 0.5 kb mgp1 fragment was cloned into the pStinger transformation plasmid. pStinger is similar to pPelican with the exception that the β-gal reporter has been replaced with nuclear EGFP. Recombination constructs were amplified with primers containing AttB sites which amplify different lengths of the mgp1 promoter. Amplified fragments were cloned into the T-vector PCR cloning vector and used for recombinatorial transformation.
(TIF)

Figure S2 **Tissue specific expression analysis of five putative homeodomain genes.** All qPCR analyses normalized to Tsetse *tub*. Error bars represent standard error. Samples represent 3 replicates of tissues isolated from 5 individuals. **A.** qPCR analysis of *nubbin (nub)* tissue specificity. **B.** qPCR analysis of *teashirt (tsh)* tissue specificity. **C.** qPCR analysis of *vismay (vis)* tissue specificity. **D.** qPCR analysis of *ladybird late* (*lbl*) tissue specificity. **E.** qPCR analysis of *pox meso (poxm)* tissue specificity.
(TIF)

Table S1 **qPCR primer sequences.** Sequences for Syber green primers utilized in the qPCR analyses performed in this work.
(DOCX)

Table S2 **Construct creation primer sequences.** Primer sequences utilized in the amplification of sequences for the creation of transgenic constructs.
(DOCX)

Table S3 **siRNA sequences.** Sequence information for siRNAs utilized within the gene knockdown experiment.
(DOCX)
