# Supplementary figures and images for: The Homeodomain Protein Ladybird Late Regulates Synthesis of Milk Proteins during Pregnancy in the Tsetse Fly (Glossina morsitans)
Source: PLoS Negl Trop Dis. 2014 Apr 24;8(4):e2645. doi: 10.1371/journal.pntd.0002645 (PMC3998940; doi:10.1371/journal.pntd.0002645)

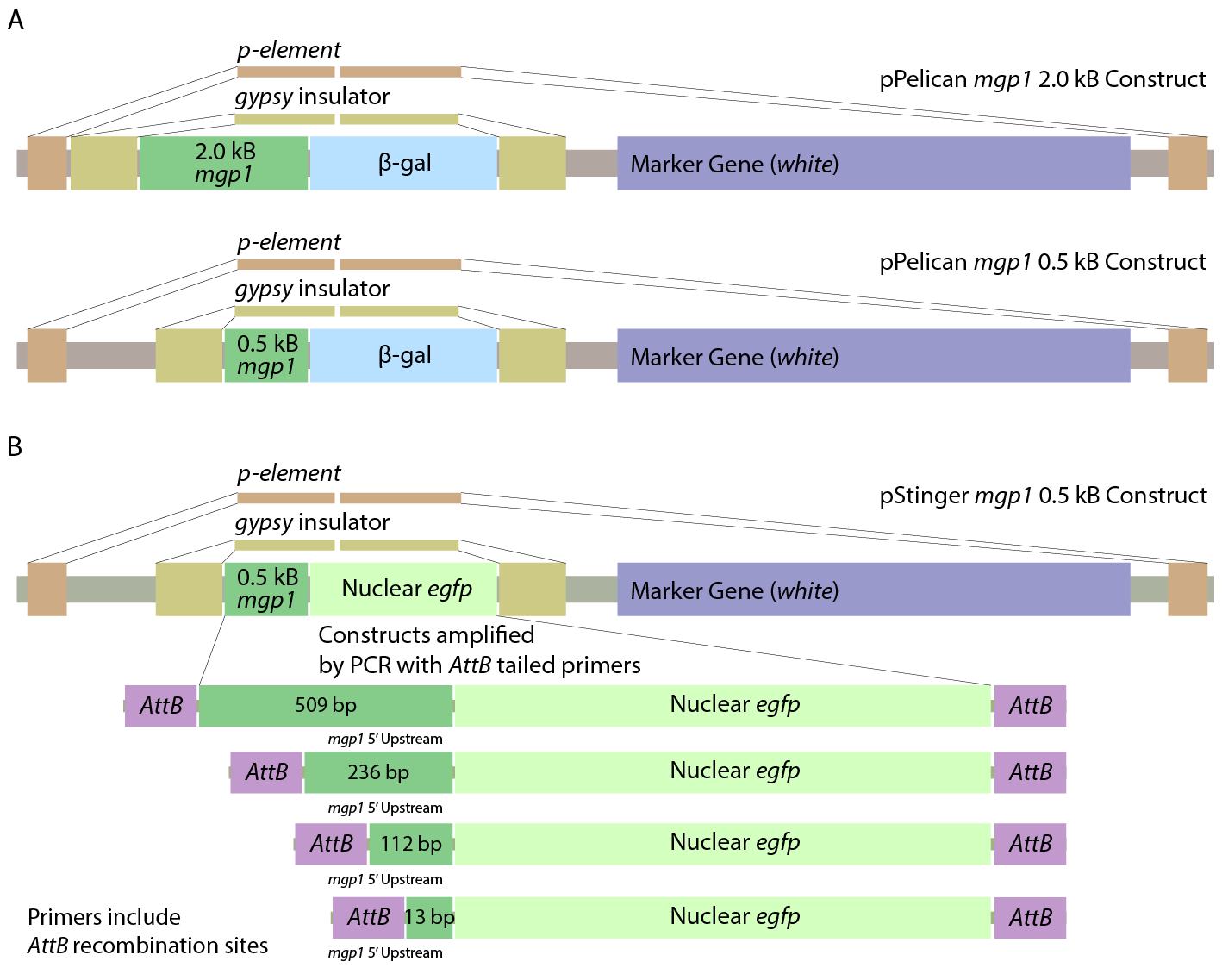

Supplement: Figure S1 — Schematic of mgp1 -reporter fusion constructs for Drosophila transformation. A. 2.0 and 0.5 kB mgp1 pPelican based transformation constructs. Constructs include 2.0 and 0.5 kB promoter fragments driving a β-gal reporter flanked by gypsy insulator sequences. The construct also includes the white transformation marker and is flanked by p-element sequences. B. 509,236,112 and 13 bp mgp1 pStinger based recombination constructs. The 0.5 kb mgp1 fragment was cloned into the pStinger transformation plasmid. pStinger is similar to pPelican with the exception that the β-gal reporter has been replaced with nuclear EGFP. Recombination constructs were amplified with primers containing AttB sites which amplify different lengths of the mgp1 promoter. Amplified fragments were cloned into the T-vector PCR cloning vector and used for recombinatorial transformation. (TIF) [file pntd.0002645.s001.tif]

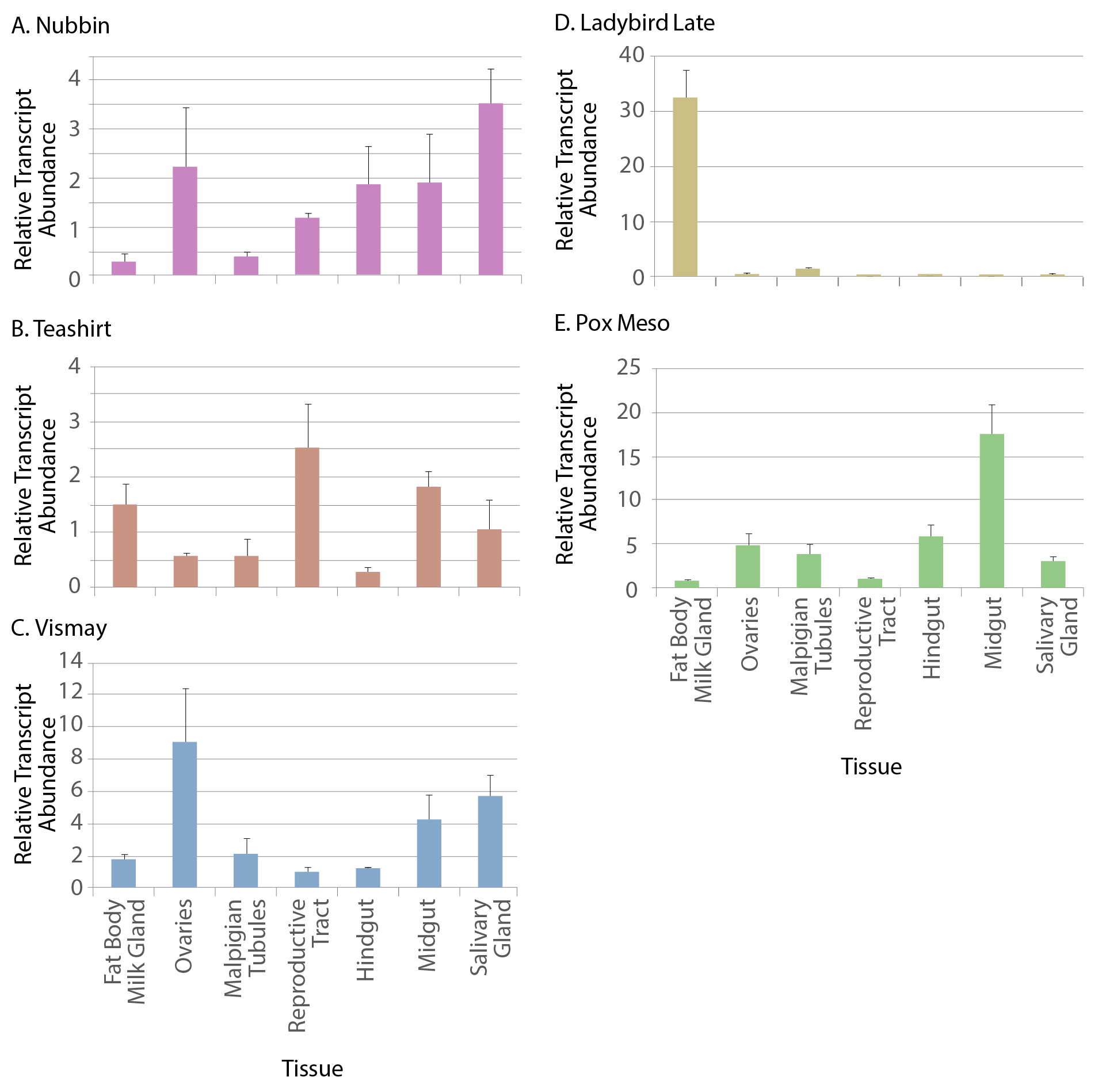

Supplement: Figure S2 — Tissue specific expression analysis of five putative homeodomain genes. All qPCR analyses normalized to Tsetse tub. Error bars represent standard error. Samples represent 3 replicates of tissues isolated from 5 individuals. A. qPCR analysis of nubbin (nub) tissue specificity. B. qPCR analysis of teashirt (tsh) tissue specificity. C. qPCR analysis of vismay (vis) tissue specificity. D. qPCR analysis of ladybird late (lbl) tissue specificity. E. qPCR analysis of pox meso (poxm) tissue specificity. (TIF) [file pntd.0002645.s002.tif]
