# Supplementary material for: The Homeodomain Protein Ladybird Late Regulates Synthesis of Milk Proteins during Pregnancy in the Tsetse Fly (Glossina morsitans)
Source: PLoS Negl Trop Dis. 2014 Apr 24;8(4):e2645. doi: 10.1371/journal.pntd.0002645 (PMC3998940; doi:10.1371/journal.pntd.0002645)
Supplement: Table S1 — qPCR primer sequences. Sequences for Syber green primers utilized in the qPCR analyses performed in this work. (DOCX) [file pntd.0002645.s003.docx]

**Table S1: qPCR Primer Sequences**

| **qPCR** | **Primer Name** | **Sequence** |
| --- | --- | --- |
| *Glossina milk protein 1* | | |
| *mgp1* | Forward | 5’-CTGGATCTTGACCCGTGAAC-3’ |
| *mgp1* | Reverse | 5’-GGGGAAGTGATGTTCCTTGA-3’ |
| *Enhanced GFP* | | |
| *egfp* | Forward | 5’- ACGTAAACGGCCACAAGTTC-3’ |
| *egfp* | Reverse | 5’- TGAACTTCAGGGTCAGCTTG-‘3 |
| *Glossina tubulin* | | |
| *tub* | Forward | 5’-CCATTCCCACGTCTTCACTT -3’ |
| *tub* | Reverse | 5’-GACCATGACGTGGATCACAG -3’ |
| *Drosophila tubulin* | | |
| *tub* | Forward | 5'-CATTACACCGAGGGTGCTG-3' |
| *tub* | Reverse | 5'-GTCAGCTGGAAGCCCTGA-3' |
| *Glossina teashirt (tsh)* | | |
| *tsh* | Forward | 5’-TCAGTATTTCGCTGCGTTTG-3’ |
| *tsh* | Reverse | 5’-TTGCTGTCCTTCATGTGAGC-3’ |
| *Glossina nubbin (nub)* | | |
| *nub* | Forward | 5’-GCAGCACCATCATCATCATC-3’ |
| *nub* | Reverse | 5’-CCACGACTTCGACCTCATTT-3’ |
| *Glossina pox meso (poxm)* | | |
| *poxm* | Forward | 5’-GGTGCCATAGGAGGTTCAAA-3’ |
| *poxm* | Reverse | 5’-TCGCTTAACAAACGATCACG-3’ |
| *Glossina vismay (vis)* | | |
| *vis* | Forward | 5’-TTGCCCAAACATTCTGTGAA-3’ |
| *vis* | Reverse | 5’-ATATTTTTCCGCATCGCTTG-3’ |
| *Glossina ladybird late (lbl)* | | |
| *lbl* | Forward | 5’-CCAACACCTTGGGACCATAC-3’ |
| *lbl* | Reverse | 5’-CGGAGAAAGGAACGGATGTA-3’ |
