# Supplementary material for: The Homeodomain Protein Ladybird Late Regulates Synthesis of Milk Proteins during Pregnancy in the Tsetse Fly (Glossina morsitans)
Source: PLoS Negl Trop Dis. 2014 Apr 24;8(4):e2645. doi: 10.1371/journal.pntd.0002645 (PMC3998940; doi:10.1371/journal.pntd.0002645)
Supplement: Table S2 — Construct creation primer sequences. Primer sequences utilized in the amplification of sequences for the creation of transgenic constructs. (DOCX) [file pntd.0002645.s004.docx]

**Table S2: Construct Creation Primer Sequences**

| **Primer Name** |  | **Sequence** |
| --- | --- | --- |
| **2 kB β-gal enhancer/reporter construct (pPelican)** | | |
| 2 kB *mgp1* promoter | Forward | 5’-GCTCTAGAATGAGTTCTTAATGAAATGATAACAAAACTAA-3’ |
| 2 kB *mgp1* promoter | Reverse | 5’- GGGGTACCGTTACTGCAAATTGACGAAGTTTTC-3’ |
| **0.5 kB β-gal and EGFP enhancer/reporter construct (pPelican and pStinger)** | | |
| 0.5 kB *mgp1* promoter | Forward | 5’- TTGTACGCCCAGAAGCCAGA-3’ |
| 0.5 kB *mgp1* promoter | Reverse | 5’- GTTACTGCAAATTGACGAAGTTTTC-3’ |
| **509 bp eGFP enhancer/reporter construct** | | |
| 509 bp *mgp1* promoter (PhiC site) | Forward | 5’-CGGGTGCCAGGGCGTGCCCTTGGGCTCCCCGGGCGCGTACGATTTTGTACGCCCA-3’ |
| 509 bp *mgp1* promoter (PhiC site) | Reverse | 5’- CGGGTGCCAGGGCGTGCCCTTGGGCTCCCCGGGCGCGTACCAACGCACACTTATT-3’ |
| **236 bp eGFP enhancer/reporter construct** | | |
| 236 bp *mgp1* promoter (PhiC site) | Forward | 5’- CGGGTGCCAGGGCGTGCCCTTGGGCTCCCCGGGCGCGTACCAGTAACAACAACAA-3’ |
| 236 bp *mgp1* promoter (PhiC site) | Reverse | 5’- CGGGTGCCAGGGCGTGCCCTTGGGCTCCCCGGGCGCGTACACTTATTACGTGAACT-3’ |
| **112 bp eGFP enhancer/reporter construct** | | |
| 112 bp *mgp1* promoter (PhiC site) | Forward | 5’- CGGGTGCCAGGGCGTGCCCTTGGGCTCCCCGGGCGCGTACCGAGCTATAAAATTTG-3’ |
| 112 bp *mgp1* promoter (PhiC site) | Reverse | 5’- CGGGTGCCAGGGCGTGCCCTTGGGCTCCCCGGGCGCGTACACGCACACTTATTAC-3’ |
| **13 bp eGFP enhancer/reporter construct** | | |
| 13 bp *mgp1* promoter (PhiC site) | Forward | 5’- CGGGTGCCAGGGCGTGCCCTTGGGCTCCCCGGGCGCGTACTGCAGTAACAATCAC-3’ |
| 13 bp *mgp1* promoter (PhiC site) | Reverse | 5’- CGGGTGCCAGGGCGTGCCCTTGGGCTCCCCGGGCGCGTACCAACGCACACTTATT-3’ |
