# Supplementary material for: The Homeodomain Protein Ladybird Late Regulates Synthesis of Milk Proteins during Pregnancy in the Tsetse Fly (Glossina morsitans)
Source: PLoS Negl Trop Dis. 2014 Apr 24;8(4):e2645. doi: 10.1371/journal.pntd.0002645 (PMC3998940; doi:10.1371/journal.pntd.0002645)
Supplement: Table S3 — siRNA sequences. Sequence information for siRNAs utilized within the gene knockdown experiment. (DOCX) [file pntd.0002645.s005.docx]

**Table S3: siRNA Sequences**

| **Gene** | **Sequence** |
| --- | --- |
| *silbl* | 5’-AUUUGCAAUCUUUCGUUGAUCAGAGCC-3’ |
| *silbl* | 5’-CGCAAAUCACGGACAGCGUUUACAA-3’ |
| *siGFP* | 5’-CUUGACUUCAGCACGUGUCUUGUAGUU-3’ |
| *siGFP* | 5’-CUACAAGACACGUGCUGAAGUCAAG -3’ |
